# Genetic structure and divergence of marginal populations of black poplar (*Populus nigra* L.) in Poland

**DOI:** 10.1038/s41598-025-86994-w

**Published:** 2025-01-23

**Authors:** Weronika Barbara Żukowska, Andrzej Lewandowski

**Affiliations:** Institute of Dendrology, Polish Academy of Sciences, Parkowa 5, Kórnik, 62-035 Poland

**Keywords:** Clones, Conservation, Gene flow, Genetic variation, *Populus nigra*, Riparian ecosystems, Biodiversity, Boreal ecology, Climate-change ecology, Conservation biology, Forest ecology, Forestry, Population dynamics, Restoration ecology, Riparian ecology, Genetic markers, Genotype, Population genetics, Biodiversity, Climate-change ecology, Conservation biology, Forest ecology, Forestry, Population dynamics, Restoration ecology, Riparian ecology, Ecology, Genetics, Ecology

## Abstract

Genetic diversity is crucial to secure the survival and sustainability of ecosystems. Given anthropogenic pressure, as well as the projected alterations connected with the level and circulation of water, riparian forests are of particular concern. In this paper, we assessed the genetic variation of black poplar – one of the keystone tree species of riverine forests. The natural habitats of black poplar have been severely transformed leading to a significant decline of its population size. Using a set of 18 nuclear microsatellites and geographic location data, we studied 26 remnant populations (1,261 trees) located along the biggest river valleys in Poland. Our main goal was to assess the overall genetic variation and to verify if range fragmentation and habitat transformation have disrupted gene exchange among populations. Genotyping revealed that 261 trees were clones. The level of clonality was higher in more transformed river sections. All populations have probably gone through a drastic genetic bottleneck in the distant past, and most of them have low effective population sizes. Still, the overall level of genetic variation remains high, but certain populations require attention due to their lower genetic variation, higher clonality and strong spatial genetic structure. Genetic differentiation was low, yet Bayesian clustering supported the existence of 11 genetic clusters. According to the results, gene exchange is most prevalent between adjacent populations. Relatively free gene flow occurs only along the Vistula, particularly in its middle section which is characterized by the highest genetic variation. Noticeable genetic structuring was observed along the Oder. Populations located at the range margin showed signs of genetic divergence and reduction of variation. We conclude that human activities have impacted the gene pool of black poplar in Poland by disrupting landscape connectivity and preventing the species from generative reproduction. The study provides practical guidelines on how to develop and implement the conservation program for the gene pool of black poplar in Poland. It also presents a strong case favoring river renaturation and genetic monitoring, particularly concerning keystone species.

**Supplementary Information:**

The online version contains supplementary material available at 10.1038/s41598-025-86994-w.

## Introduction

Trees, as keystone species of forest ecosystems, are of great importance for maintaining biodiversity while simultaneously fulfilling multifaceted functions. In the era of climate change, the critical importance of forests in mitigating the adverse impacts of environmental shifts is invaluable^1^. Forest trees, with their limited dispersal capacity, late reproductive maturity and prolonged lifespans, face particular challenges in coping with climate change. To survive, they are forced to adapt, either via plastic responses or genetic changes. Therefore, recognizing genetic variability remains essential for safeguarding species and populations and thus maintaining the stability of ecosystems^2^. In accordance with the central dogma of conservation genetics, “Genetic variability is beneficial, hence worth preserving to the greatest extent”^3^. The priority should be given to populations at range margins as they are likely to hold genetic variants critical for adaptation to changing climate^4^.

Most studies of genetic variation and genetic monitoring programs of forest tree species focus on economically important taxa, primarily those used in the timber industry. From the point of view of biodiversity conservation, tropical forests have been the center of attention for decades and are still considered the most valuable in this respect, being extremely threatened at the same time^5,6^. Meanwhile, it appears that riparian forests harbor the highest species richness among forests, both at local and regional scales^7,8^. Moreover, they provide a variety of ecosystem services^9^ and constitute natural corridors that connect larger forest areas^9,10^. Unfortunately, the high productivity of riverine habitats has led to extensive biological impoverishment of these areas due to their overexploitation. It is estimated that about 99% of riparian forests in Europe have already disappeared^11,12^. Moreover, the impacts of climate change on these unique habitats are likely to be particularly significant, given the anticipated alterations in snowpack and hydrological patterns^13^.

The most common tree species found in riparian forests vary depending on the region and local conditions. Typically, riverine areas are inhabited by a mix of broadleaves adapted to the specific hydrological conditions and ecological context of each region. Common species found in Europe include willows, poplars, birches and alders^14^. Some species are found exclusively in floodplain areas such as black poplar (*Populus nigra* L.) which forms natural poplar communities known as the *Salici-Populetum* (willow-poplar forest) or *Populetum albae* (poplar forest). Black poplar is distributed across Eurasian riparian ecosystems, spanning from Morocco and Ireland in the west, across Russia and to China in the east. As a pioneer taxon with a preference for sunlight, it predominantly establishes populations by colonizing open spaces on alluvial soils through various means such as seeds, cuttings or root fragments^15^. Black poplar is known for its rapid growth and ease of vegetative propagation. It also appears to be highly plastic and resistant to environmental pollution. For these reasons, this species is often used for soil protection and phytoremediation purposes in polluted areas^16^. Black poplar has been extensively crossed, facilitating the development of new, resilient poplar varieties used to establish short rotation tree plantations^16–18^.

The viability of black poplar populations heavily relies on fluvial dynamics, especially on periodic flooding events, which are essential for the natural regeneration of this species^19^. Although black poplar produces large amounts of seeds, which are easily transported by wind and water, they are short-lived and very light^20^. Most importantly, they require specific environmental conditions and bare, moist surfaces for germination and further growth^20,21^. In nature, such conditions occur only as a consequence of late spring floods when the seeds are released. Human activities like wetland drainage, flood control measures, deforestation and intensive land use along riverbanks have altered flooding dynamics and caused the loss of natural habitats of black poplar. Therefore, this tree now faces the threat of extinction and is considered endangered in many European countries^10,11^.

Several research groups have investigated the genetic diversity of black poplar along river valleys in Western and Southern Europe^22–28^. The results showed that most populations, though fragmented, still maintain a high level of genetic variation. Other studies have focused on the clonality of black poplar as well as sex bias^28–30^. It is also important to mention that black poplar naturally hybridizes with artificially introduced Euramerican hybrids (*P.* × *canadensis* Moench, i.e. *P.* × *euramericana*) and varieties of *P. nigra* such as Lombardy poplar (*P. nigra* cv. ‘Italica’ Duroi). This phenomenon may pose a significant threat to the genetic purity of black poplar. The rate of introgression seems to be rather low^25,31^, yet some studies found it very high, up to nearly 50%^32^. The results strongly depended on the research area and methods used.

Black poplar is not legally protected in Poland. Its populations are becoming smaller and most of the trees are at advanced age and in poor health condition. Larger groups of individuals still exist along major river valleys, but the species reaches its distribution limit in the north of the country. Natural regeneration through sexual reproduction occurs almost exclusively in the middle section of the Vistula, which is the biggest river in Poland. The worst situation can be observed along the two most transformed river valleys, i.e. the Oder and Warta. The first studies of the genetic diversity of black poplar in Poland were carried out for two closely located stands in the middle section of the Vistula^33,34^. They were characterized by a high level of genetic diversity^34.35^, but a homogeneous gene pool^34^. Additionally, there were some hybrid individuals among the one-year-old seedlings^34^. More comprehensive research is limited only to the Oder valley, where Wójkiewicz et al.^35^ detected clear signals of genetic erosion accompanied by rather high clonality.

In this study, we used nuclear microsatellites and geographic location data for 26 remnant black poplar populations located along the biggest river valleys in Poland. We aimed to analyze the gene pool composition of each stand as well as their spatial genetic structure (SGS). Specifically, we wanted to answer the following questions: (1) What is the level of genetic variation of black poplar in Poland, and is it comparable to other areas, especially in the case of marginal stands? (2) Is genetic differentiation lower within the same river catchment than between them? (3) Are constraints on gene flow greater along more transformed river sections? (4) What is the strength and distribution of SGS in each population? (5) Does the gene pool of black poplar in Poland require protection and how it should be done?

## Materials and methods

### Study sites and DNA extraction

**Fig. 1:**
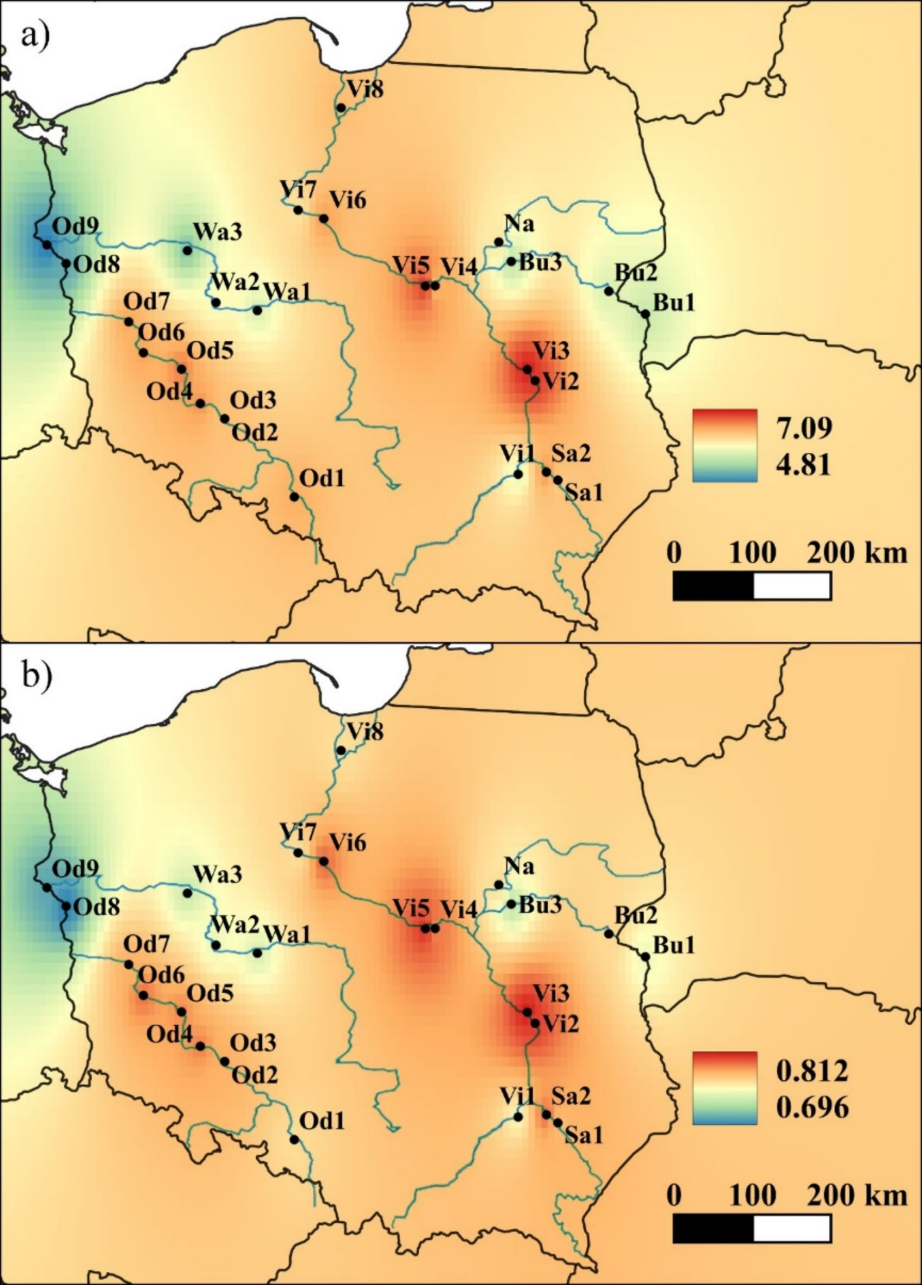
IDW interpolation of: (**a**) allelic richness values (*A*_R_); (**b**) expected heterozygosity values (*H*_E_). The map was generated using QGIS software, version 3.22.7 [https://qgis.org].

The analyses were carried out for 26 black poplar populations located along the six biggest river valleys in Poland, i.e. the Vistula (8 populations), Oder (9), Warta (3), Bug (3), Narew (1) and San (2), yielding a total of 1,261 trees (Table S1, Fig. 1). We tried to incorporate populations from the upper, middle and lower sections of all rivers to maximize environmental amplitude and cover the species distribution range as even as possible. We checked the geographic location of each tree, measured the diameter at breast height of all individuals to estimate their approximate age and collected leaves for DNA extraction. For most locations, we sampled all individuals. In the case of a few larger and scattered populations (i.e. Od1, Od2, Od3, Sa2), we focused on the area with the highest tree density, from which we sampled all trees. Leaf tissue was stored at -20 °C. DNA was extracted from 100 to 120 mg of frozen tissue ground in liquid nitrogen using the CTAB protocol^36^ with an additional step which involved mixing the solution with chloroform and centrifuging it before adding isopropanol to the upper phase. The quality of the extracts was checked using 1% agarose electrophoresis. The DNA concentration was measured with a BioPhotometer plus spectrophotometer (Eppendorf AG, Germany) and adjusted to 15–20 ng/µl.

### Molecular analyses

After an initial stage of marker testing, we chose 18 polymorphic nuclear microsatellites^37,38^ for which the genotyping results were of good quality, unambiguous and repeatable. The frequency of null alleles did not exceed the level of 0.19, as recommended by Chapuis et al.^39^ The selected markers were amplified in three multiplex PCRs following the protocols described by Wójkiewicz et al.^33^ Fluorescently labeled amplification products were genotyped using an ABI3130*xl* capillary sequencer with GeneScan™ 500 LIZ™ internal size standard (Thermo Fisher Scientific, USA). Raw data were scored with GeneMapper ver. 4.0 (Thermo Fisher Scientific, USA) and verified manually. Binning of alleles was carried out using Tandem^40^ and adjusted manually. To account for allelic drift between particular runs, we incorporated a set of known genotypes in each run. We also made sure that our dataset did not include cryptic hybrids using a set of molecular markers as well as diagnostic microsatellite loci, as described by Wójkiewicz et al.^35^.

### Parameters of genetic diversity

We first used RClone package^41^ for R to estimate the number of clonal lineages, because slightly different genotypes may be the same genet, either due to genotyping errors or somatic mutations. Ramets within each genet were excluded from further analyses; therefore, the final dataset consisted of only one repetition of each genotype. The calculations of genetic variation parameters were carried out for each population and river in GenAlEx ver. 6.5^42,43^. These included the mean number of alleles (*A*), mean effective number of alleles (*A*_E_), number of private alleles (*A*_P_), mean observed (*H*_O_) and expected heterozygosity (*H*_E_). Mean rarefied allelic richness (*A*_R_) was computed with FSTAT ver. 2.9.4^44^. To account for the presence of null alleles, we adjusted the inbreeding values (*F*_IS_) with a Bayesian approach implemented in INEst ver. 2.2^45^. We set 200,000 Monte Carlo cycles, updates performed every 20th cycle and a burn-in of 20,000. The significance of inbreeding was assessed based on the comparison of the deviance information criterion (DIC) calculated for the models with and without inbreeding as described in the INEst manual.

### Population differentiation and genetic structure

The level of genetic differentiation was assessed using two commonly used differentiation indexes: fixation index (*F*_ST_) and Slatkin’s analog of *F*_ST_ (*R*_ST_). The values of both indexes were calculated in Arlequin ver. 3.5.2.2^46^ for all pairs of populations and rivers. We used the same software to run the analysis of molecular variance (AMOVA). AMOVA was conducted between the Oder and Vistula catchments, as well as for each catchment separately, dividing them by rivers and populations. Subsequently, we tested whether the genetic similarity among populations decreases with distance (IBD – isolation by distance) with the Mantel test implemented in GenAlex. GenAlEx was also used to carry out the principal coordinates analysis (PCoA) to visualize the genetic relationships among the studied black poplar populations. In each case, p-values were calculated with 10,000 permutations by permuting haplotypes at the level of populations and catchments or populations at the level of rivers.

Population genetic clustering was performed using STRUCTURE^47^ with 100,000 iterations following a burn-in of 10,000. We opted for the admixture ancestry model with correlated allele frequencies because it gives more reliable results when the population structure is subtle^48^. Ten runs were set for a number of genetic clusters (K) ranging from one to 15. The most probable K number was determined based on the Evanno^47^ and Puechmaille^49^ methods as the latter is supposed to be more reliable when the sampling is uneven. We chose two thresholds for the Puechmaille method by setting the membership coefficient to 0.5 and 0.7. Finally, we used StructureSelector web-based software^50^ to align multiple runs for the optimal K value with the LargeKGreedy algorithm and to generate admixture bar plots.

### Spatial autocorrelation

The spatial autocorrelation was examined separately for each population. For this purpose, we first used SPAGeDi ver. 1.5^51^ to calculate the average Loiselle’s kinship coefficients^52^ for pairs of individuals within each location. Distance intervals were adjusted to get a similar number of pairs in ten distance classes. We then created correlograms by plotting the average kinship coefficients against ten distance classes. The autocorrelation was considered significant if the observed data fell outside the 95% CIs. The strength of SGS was quantified with the Sp statistics which equals -(b-log)/(1-F1), where b-log is the slope of the regression of pairwise kinship coefficients on the natural logarithm of the spatial distance^53^. In each case, p-values were calculated with 10,000 permutations.

### Demographic history

To assess the risk caused by genetic drift and inbreeding depression, we calculated effective population sizes (Ne) with the LD approach^54^ available in NeEstimator ver. 2.01^55^. The allele frequency cutoff (P_crit_) was set to 0.05. 95% CIs were calculated using the parametric option with χ2 approximation. We further tested the probability of past bottlenecks with two approaches, both available in INEst. The first test was developed by Cornuet and Luikart^56^. It assumes that a recent bottleneck results in an excess of heterozygotes compared to a population with a constant size. Following the parameters recommended by Peery et al.^57^, we conducted this test with 10,000 coalescent simulations. A two-phase model (TPM) was applied to incorporate both single-step and multistep mutations, which is considered best for microsatellite data^58^. The second approach was proposed by Garza and Williamson^59^ and is known as the test for the deficiency in M-Ratio (MR). It is supposed to detect more severe bottlenecks occurring in the distant past^60^. The simulation of a demographically stable population (MR^eq^) was determined as the mean of 10,000 coalescence replicates. For both bottleneck tests, we applied the Wilcoxon signed-rank test to compute p-values with 1,000,000 permutations.

## Results

### Clonality and genetic variation

Clonality analysis revealed that 261 individuals were clones and they were present in 22 out of 26 populations (Table S1). This constitutes 20.70% of all trees examined. Duplicate genotypes did not occur only in two populations from the middle Vistula (Vi3 and Vi4) and in single locations from the San (Sa2) and Bug (Bu3). Clonal genotypes were replicated two to six times and were in close proximity. The level of clonality, however, varied greatly between individual populations. The highest values were in the upper section of the Vistula River (pop Vi1) and in the marginal population Vi8 (Table S1). In general, populations growing along highly transformed river sections (Oder, Warta, upper and lower Vistula) had higher levels of clonality compared to those located in less transformed regions (middle Vistula, San, Bug, Narew) (*p* < 0.05 in a one-sided Student’s t-test). Genotypic richness for individual locations ranged from *R* = 0.26 to *R* = 1.00 (Table S1), while the average values for rivers ranged from *R* = 0.72 to *R* = 0.98 (Table 1).

**Table 1:** Basic genetic variation parameters calculated for each river valley.

River	*N*_*P*_	*N*_I_	*N*_L_	*R*	*A*	*A*_*R*_	*A*_E_	*A*_*P*_	*H*_O_	*H*_E_
Oder	9	536	388	0.72	17.22	11.44	7.32	21	0.721	0.808
Warta	3	151	117	0.77	12.78	9.85	5.32	4	0.734	0.782
Vistula	8	321	247	0.77	18.11	11.60	7.45	30	0.731	0.815
San	2	95	93	0.98	13.00	10.48	6.50	5	0.757	0.797
Bug	3	127	125	0.98	12.44	9.51	5.41	1	0.704	0.776
Narew	1	31	30	0.97	8.89	8.78	5.29	1	0.703	0.767
Total	26	1261	1000							
Mean	~ 4	~ 210	~ 167	0.87	13.74	10.28	6.22	~ 10	0.725	0.791

*N*_P_ – Number of populations; *N*_I_ – Number of individuals; *N*_L_ – Number of clonal lineages; *R* – Genotypic richness (*N*_L_/*N*_I_); *A* – Mean number of alleles; *A*_R_ – Mean rarefied allelic richness; *A*_E_ – Mean effective number of alleles; *A*_P_ – Number of private alleles; *H*_O_ – Mean observed heterozygosity; *H*_E_ – Mean expected heterozygosity.

The mean number of alleles calculated for rivers equaled *A* = 13.74 and it was higher than the mean value of allelic richness (*A*_R_ = 10.28) (*p* < 0.05 in a one-sided Student’s t-test). Due to the presence of many rare and private alleles, the mean effective number of alleles was much lower (*A*_E_ = 6.22). The number of private alleles was a few times higher in the two longest rivers (the Vistula and Oder, *A*_P_ = 30 and *A*_P_ = 21, respectively), with the mean value of *A*_P_ = ~ 10 (Table 1). At the population level, however, we could observe slightly lower values of allelic variation along the Warta and Bug rivers, as well as in the Narew population. In most cases, the populations from the lower section of the river had the lowest values of allelic variation parameters, especially Od8 and Od9. Population Vi1 appeared the least variable along the Vistula. On the other hand, the highest genetic variation was detected in the middle section of the Vistula. The level of heterozygosity was high and comparable in all populations (mean *H*_O_ = 0.720, range: 0.654–0.775; mean *H*_E_ = 0.769, range: 0.696–0.812), though a bit lower in Od8, Od9 and Vi1 (Table S1, Fig. 1).

### Genetic differentiation

The results of AMOVA indicated very low but significant genetic differentiation between the Oder and Vistula catchments (*F*_ST_ = 0.018; *R*_ST_ = 0.028). The differentiation between the Oder and its tributary, the Warta, was at a similar level, but only for *F*_ST_, which was 0.025. For the Vistula and its tributaries, both *F*_ST_ and *R*_ST_ values were statistically insignificant. Within both catchments, we found that differentiation among populations was around 5% (Table 2). The pairwise *F*_ST_ values were higher between pairs of rivers from different catchments compared to those within the same catchment (*p* < 0.05 in a one-sided Student’s t-test). It is worth noting that in the case of *R*_ST_, no significant differentiation was found between the Narew and Oder, as well as the Narew and Warta (Table 3). Comparisons between populations showed that Od8, Od9 and Wa3 were the most genetically different from other locations, with the values of pairwise *F*_ST_ as high as 0.142 and even 0.252 for *R*_ST_. High pairwise *R*_ST_ values were characteristic for population Vi1 (Table S2). The Mantel test showed a low but significant correlation between geographic and genetic distances among the populations (R^2^ = 0.144; *p* < 0.05) (Fig. S1).

**Table 2:** Results of AMOVA.

			Variation	
Catchment	Source of variation	d.f.	*F*_ST_	*R*_ST_
	Among catchments	1	0.018	0.028
	Within catchments	1998	0.982	0.972
Oder	Among rivers	1	0.025	*0.001*
	Among populations	10	0.049	0.054
	Within populations	998	0.926	0.945
Vistula	Among rivers	3	*0.008*	*0.011*
	Among populations	10	0.036	0.050
	Within populations	976	0.956	0.939

Values that are not significant (*p* > 0.05) are written in italics. d.f. – degrees of freedom.

**Table 3:** Pairwise *F*_ST_ (below diagonal) and *R*_ST_ (above diagonal) values among the studied populations grouped into rivers.

	Oder	Warta	Vistula	San	Bug	Narew
Oder		0.014	0.030	0.050	0.059	0.014
Warta	0.037		0.021	0.064	0.065	*0.008*
Vistula	0.017	0.035		0.043	0.020	*0.004*
San	0.029	0.054	0.012		0.038	0.053
Bug	0.039	0.060	0.020	0.031		0.043
Narew	0.056	0.065	0.029	0.043	0.036	

Values that are not significant (*p* > 0.05) are written in italics.

### Population structure

**Fig. 2:**
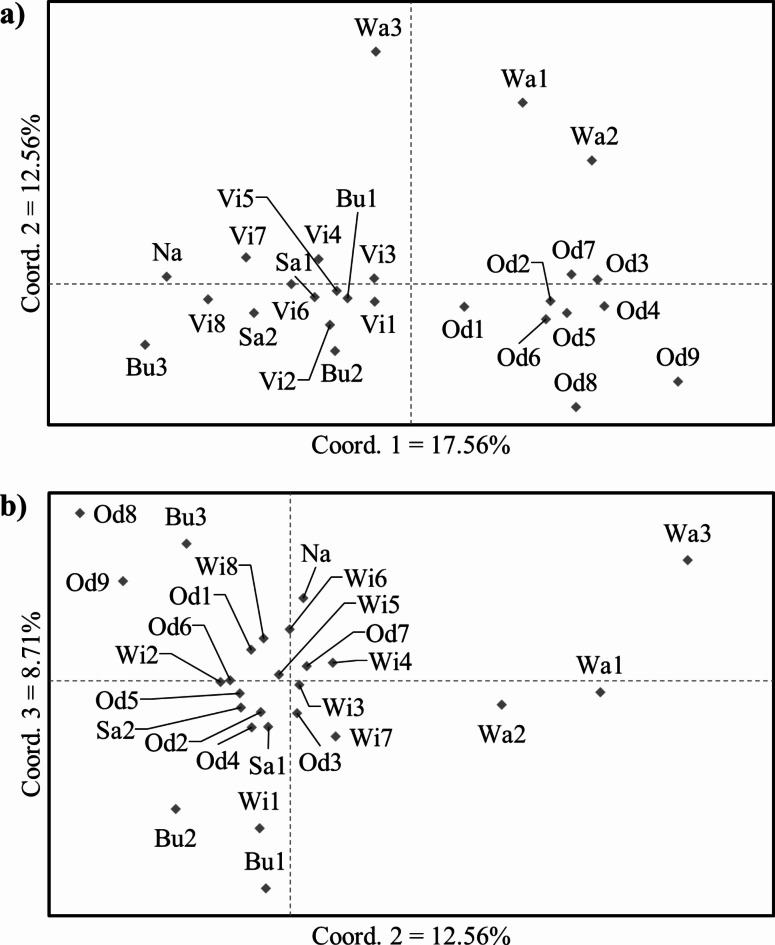
Results of PCoA showing: (**a**) coordinate 1 vs. 2; (**b**) coordinate 2 vs. 3.

According to the results of the PCoA, the first two coordinates explained 30.12% of the total genetic variation. We could distinguish two larger groups of populations. The first group comprised the Vistula and its tributaries which largely shared the same gene pool. Only populations Bu3 and to a lesser extent Na were slightly genetically different. All populations from the Oder could be assigned to the second genetic group. All three Warta populations were outliers, especially Wa3 (Fig. 2a). The third coordinate explained an additional 8.71% of the total genetic variation. When we plotted coordinates 2 vs. 3, the Warta populations remained outliers. Most populations were clustered around average values of both coordinates. However, the third coordinate separated populations Od8, Od9 and Bu3 (on one side) and Wi, Bu1 and Bu2 (on the opposite side) (Fig. 2b).

**Fig. 3:**
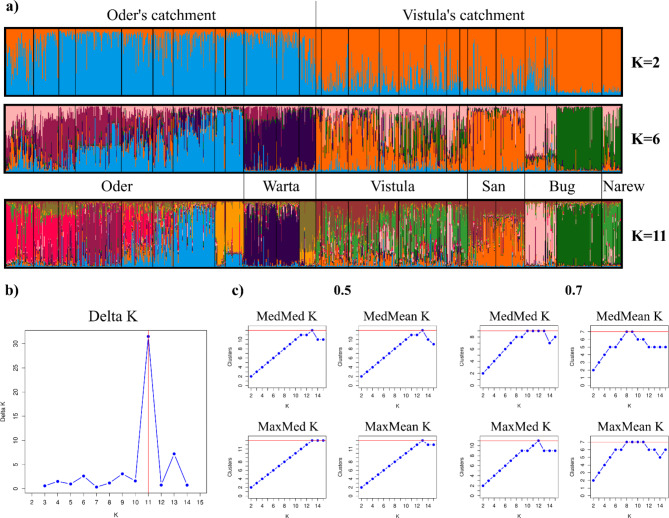
Results of Bayesian clustering: (**a**) barplots of the STRUCTURE results for K = 2 (two river catchments), K = 6 (six rivers) and K = 11 (most probable number); the order of the populations corresponds to Table 4; (**b**) distribution of ΔK according to the Evanno method; (**c**) four Puechmaille estimators (MedMed K, MedMean K, MaxMed K, MaxMean K) calculated with membership coefficients set to 0.5 and 0.7.

The results of Bayesian clustering showed that populations occupying two river catchments (Oder and Vistula) had two distinguishable gene pools with varying degrees of admixture. Only populations Bu3 and Na had a negligible level of admixture (Fig. 3a). For K = 6, which corresponds to the number of rivers, there was a noticeable difference between the gene pools of the Oder and Warta rivers. Along the Oder, there was a progressive change in the proportions of different genetic clusters. We observed the highest admixture along the Vistula valley. Additionally, there was a high similarity between the gene pools of the populations from the San River (Sa1, Sa2) and the upper Vistula (Vi1, Vi2). The Bug River could be divided into two genetic groups: the first group (Bu1, Bu2), which had a gene pool partially similar to that of the Vistula’s populations; and the second group (Bu3), which shared a significant proportion of its gene pool with the Narew (Na) (Fig. 3a). The most probable number of distinct genetic clusters identified with the Evanno method was K = 11 (Fig. 3b). When we chose the Puechmaille method, the results differed for the two membership criteria, from 12 to 13 clusters when the membership coefficient was set to 0.5, to 7–11 clusters with a more stringent 0.7 criterion (Fig. 3c). Therefore, we considered K = 11 as the most probable cluster number. Grouping at K = 11 revealed further subtle substructure where Od4, Od8 with Od9 and Wa3 formed three recognizable subgroups (Fig. 3a).

### Inbreeding and SGS

The comparison of full and random mating models indicated that inbreeding was significant in ten locations. Nevertheless, the statistically significant values of the inbreeding coefficient were low and ranged from *F*_IS_ = 0.003 to *F*_IS_ = 0.027 (Table 4). We detected significant SGS in most populations. The highest and statistically significant degree of relatedness was found in the first two distance classes in the case of 14 populations. A particularly strong correlation was revealed in Od8, Od9, Wa3 and Vi1 (Sp = 0.058–0.088; *p* < 0.05). The average distance between individuals at which genetic structure was statistically significant was ~ 50 m in populations from the Vistula and Narew. For two San and most Oder populations, the kinship coefficient progressively decreased even at longer distances (up to ~ 80–120 m). On the other hand, there was no SGS in all populations from the Bug (Fig. S2).

**Table 4:** Inbreeding coefficients, effective population sizes and M-Ratios calculated for each population.

River	Pop	*F*_IS_	Ne	Ne CI95	MR	MR^eq^
Oder	Od1	0.015	27	24–32	0.617	0.780
	Od2	0.006	32	27–38	0.582	0.778
	Od3	0.007	20	17–24	0.602	0.759
	Od4	0.007^ns^	23	21–25	0.666	0.799
	Od5	0.007^ns^	86	67–117	0.665	0.781
	Od6	0.004	34	28–43	0.576	0.764
	Od7	0.010^ns^	42	38–48	0.667	0.797
	Od8	0.026^ns^	6	5–8	0.516	0.778
	Od9	0.007^ns^	6	5–7	0.507	0.805
Warta	Wa1	0.027	27	24–32	0.610	0.796
	Wa2	0.009^ns^	129	82–275	0.611	0.777
	Wa3	0.027^ns^	9	7–10	0.477	0.793
Vistula	Vi1	0.010	15	10–28	0.520	0.702
	Vi2	0.026^ns^	325	153-∞	0.626	0.772
	Vi3	0.019^ns^	89	71–117	0.664	0.774
	Vi4	0.077^ns^	18	15–21	0.577	0.769
	Vi5	0.006	115	83–179	0.661	0.773
	Vi6	0.007^ns^	32	27–40	0.598	0.760
	Vi7	0.007^ns^	11	9–14	0.596	0.752
	Vi8	0.045^ns^	231	148-∞	0.590	0.709
San	Sa1	0.007^ns^	22	19–25	0.660	0.784
	Sa2	0.003	39	33–46	0.629	0.783
Bug	Bu1	0.005	21	18–26	0.594	0.785
	Bu2	0.014^ns^	38	26–65	0.598	0.756
	Bu3	0.008	45	38–52	0.625	0.809
Narew	Na	0.021	31	26–39	0.588	0.775

Pop – Population acronym; *F*_IS_ – Inbreeding coefficient corrected for the presence of null alleles; Ne – Effective population size; MR – Observed M-Ratio; MR^eq^ – M-Ratio under mutation-drift equilibrium; ^ns^ – Not significant.

### Demographic history

We found that effective population sizes significantly differed but they were low in most stands (mean Ne = 31; range: 6-325). Low and similar effective population sizes were calculated for the populations from the Oder, San and Bug, whereas in the case of the Warta and Vistula the Ne values were very variable. The test for the excess of heterozygotes turned out not significant in any case. Therefore, the results did not support any recent bottlenecks. On the contrary, the calculated MRs were all significantly lower than MRs^eq^, indicating that all populations went through a drastic bottleneck in the distant past (Table 4).

## Discussion

### Genetic resources of black poplar in Poland

Our study revealed that the genetic resources of black poplar in Poland are still rich; however, some populations, especially those closest to the northern range limit, exhibited reduced genetic variability. Life form and breeding system greatly influence genetic diversity and its distribution^61^. As a result, woody plants have very high genetic variation, accompanied by low interpopulation differentiation^61^. Though the natural riparian habitats of black poplar have been largely transformed, leading to the high reduction and fragmentation of native populations, studies using molecular markers showed that the remnants of these populations still maintain a high level of genetic variation. The results of our study are in line with these observations when compared to previous reports from Western and Southern Europe, with the caveat that comparisons between studies should be made cautiously due to potential biases introduced by different sets of markers. Smulders et al.^24^, who analyzed the genetic variation of 17 black poplar populations along seven river catchments, found that the average level of observed heterozygosity was *H*_O_ = 0.740, compared to *H*_O_ = 0.725 in our study. The mean number of alleles was *A* = 15.71, compared to 13.74 in our study. Research carried out along a short distance of ca. 5 km in the Morava River valley (Czech Republic) revealed a slightly higher mean *H*_O_ = 0.800 but a similar mean *H*_E_ = 0.820^23^ (vs. mean *H*_E_ = 0.791 in our study). The values of *A* and *A*_E_ were also comparable (mean *A* = 11.00 vs. *A* = 13.74 and mean *A*_E_ = 6.01 vs. *A*_E_ = 6.22)^23^. Four Serbian populations had mean values of *A* = 10.29, *H*_O_ = 0.703 and *H*_E_ = 0.808^26^. Similar values of heterozygosity were also detected in 12 populations growing along the Danube River, which extends from Romania to Germany (mean *H*_O_ = 0.695, *H*_E_ = 0.811). Only allelic variation was lower (mean *A* = 8.96). This was most probably caused by the lower number and less polymorphism of microsatellite markers used in that research^25^. Our results confirm previous findings of studies conducted along the Oder^35^ and in the middle Vistula^33,34^.

In contrast, a lower level of mean *H*_E_ = 0.560 was detected in five black poplar populations located along two highly fragmented river ecosystems in Turkey^27^. Values of *A* = 4.27 and *A*_E_ = 2.40 were also considerably lower than in other river catchments in Europe, including our study. At the same time, *H*_O_ = 0.820, and the authors observed a significant excess of heterozygotes. They explained these results with possible recent bottlenecks which resulted in the excess of heterozygosity – which was not the case in our study. Alternatively, elevated *H*_O_ might be the consequence of human-mediated artificial selection which promoted superior trees that were highly heterozygous^27^.

It is important to note that the number of alleles and heterozygosity depend on the set of markers used. The aforementioned previous studies were conducted using sets of microsatellites that largely, though not entirely, overlapped with the set of markers used in our research.

Despite the overall high genetic variation of black poplar in Poland, it has to be noted that some populations displayed lower values of genetic variation parameters. This was particularly true for the lowest section of the Oder, but signs of genetic depletion were also visible along the Warta and Bug rivers. Habitat transformation, which has contributed to the decrease in effective population sizes, might be the main driver of this situation. Also, the distance separating Od8 and Od9 from other Oder populations is larger. It seems that it is too far for effective gene exchange. Moreover, Od8, Od9 and Wa3 populations are located at the range margin, making them more prone to the adverse effects of genetic drift^62^.

### Variations in the level of clonality

The level of clonality varied among locations, with the percentage of clones ranging from 0 to 74%. It was higher in populations growing in more transformed river sections. Earlier reports showed that clonality in black poplar is indeed variable. For example, Arens et al.^63^ reported that the level of clonality in two older populations occupying the Rhine river banks in the Netherlands was at least 82%. Chenault et al.^30^ found out that more than 50% of genotypes were replicated within a 7 ha area along the Loire River in France. On the other hand, the analysis of five black poplar populations in Turkey revealed a much lower share of clones (12% of all trees)^27^. Though in most studies clones were spatially restricted, in some cases they stretched even for several kilometers, but these trees were most probably planted^24^. Sprouting and root suckering in black poplar occur after direct damage, either to the shoot or root system^29^. Therefore, the most likely causes of clonal propagation of black poplar include flood training, beaver activity and mowing^29,64,65^. In natural populations, the likelihood of vegetative regeneration depends on habitat conditions, surrounding vegetation and the nature and intensity of disturbance. It is common, though, that only some genets spread vegetatively. Such situation may depend on both extrinsic (ecological) and intrinsic (genetic) factors^66^. It seems that one of the reasons for elevated clonality is the regulation of rivers^24,27,35^. Our results are in line with this suggestion, as the frequencies of clones were higher along the Oder and Warta rivers, as well as in the upper and lower sections of the Vistula.

At the level of populations, high clonality was detected in Vi1 and Vi8. When it comes to Vi1, this population comprises trees belonging to several age classes that grow at some distance from the current river course. We observed strong SGS at this location, but currently, there is no possibility of regenerating by seeds because of the high grass vegetation, poor light availability and competing tree species. Many younger individuals have likely grown from root suckers, resulting in a high number of clonal replicates occurring close to each other. This hypothesis can be further supported by the fact that duplicated genotypes were spatially limited. Vi8 is a small population, situated even further from the river current than Vi1, in the area of an old river bed. This is most likely the northernmost stand of black poplar in Poland, located at the species distribution limit. Clonal reproduction is frequently more prevalent at range margins^66^. The trees from Vi8 grow in a dense forest and the youngest are ca. 30–40 years old with a few individuals being more than 100 years old. There is a considerable number of clones that might have grown from root suckers, root stumps and trunks of fallen individuals. Nevertheless, in some cases, the distance separating duplicated genotypes was several dozen meters. Therefore, it is possible that at least some trees were planted. The planting material may have been obtained from various sources, which would be consistent with a rather high Ne = 231 of this population and the lack of SGS. What is more interesting, the gene pool of Vi8 was unique, partly due to the presence of one private allele at the WPMS04 locus with a relatively high frequency of 19.20%. Microsatellites are supposed to be selectively neutral. Therefore, this is likely the consequence of genetic drift and bottlenecks^67^ or the mentioned possibility that some part of this population has an artificial origin.

Higher clonality lowers the genetic diversity of a particular population^66^ and presumably decreases its long-term adaptability. Nevertheless, the effective number of alleles and heterozygosity should increase over time^68^. Furthermore, the time between generations is delayed which ultimately leads to the lower rate of the loss of rare alleles through genetic drift^69^. Elevated clonality is also connected with the so-called “persistence niche”^70^. The prevalence of vegetative propagation is believed to enable the persistence of local populations when the sites available for colonization are not suitable for seedling recruitment (spatially and temporally)^30^. Taking into account climate change, as well as habitat disturbances caused by humans, we may observe a gradual transition in the reproduction strategy of black poplar and other woody sprouters, changing from a generative to a vegetative one.

### Genetic structure and constraints in gene flow

Our study revealed low genetic differentiation among the Polish black poplar populations. Despite this, we observed some genetic substructure along the Oder, and, to a lesser extent, along the Warta and Bug rivers. Low interpopulation differentiation of black poplar has also been found in other European regions. For example, Čortan et al.^26^, who analyzed three Serbian populations, showed that 4.67% of genetic variation may be attributed to interpopulation variation, which is no different from our study. Nevertheless, this research covered a smaller area and comprised only 120 trees. Smulders et al.^24^ detected slightly higher genetic differentiation (8.10%), but it was calculated for populations from seven river catchments spanning Southern and Western Europe. Differentiation within particular rivers was not significant for two rivers, and around 1.8% for the remaining ones, except for the Danube (3.66%)^24^. The differentiation found among black poplar populations appears to occur partly due to the IBD. This observation is consistent with the Mantel test result indicating a weak, positive correlation between geographic and genetic distances in our study (R^2^ = 0.144; *p* = 0.010). Significant IBD and differentiation slightly above 4% was also found by Imbert and Lefèvre^22^ along the Drôme River in France.

Wind-mediated gene flow can potentially occur at long distances. In the absence of physical constraints, it may lower genetic differentiation among populations, even when they are located in different river catchments^24,26^. In this study, however, we found that despite low genetic differentiation and partial admixture of the gene pools between the Oder and Vistula catchments, they form two noticeable genetic groups. Furthermore, there is genetic substructure within both catchments, which is more evident in the transformed valleys of the Oder and Warta compared to the Vistula and its tributaries. This indicates constraints in gene flow, likely caused by habitat fragmentation and riverbed regulation. Our findings support earlier reports by Wójkiewicz et al.^35^, as well as previous studies carried out in transformed riparian areas^24,26,27^. Regarding populations Od8 and Od9, their gene pools seem to be impoverished compared to other locations. This makes them particularly susceptible to the adverse effects of genetic drift. As indicated by the results of the PCoA and Bayesian clustering, all three populations from the Warta were outliers. The gene pool of Wa3 was particularly distinct. In the transformed ecosystems of Warta, the natural regeneration of black poplar faces significant challenges. This is primarily due to the long-term impact of the Jeziorsko lake reservoir, constructed in 1968 at the upper section of the Warta River, spanning over 42 km^2^. The establishment of this reservoir has greatly minimized flooding, rendering sexual reproduction of black poplar nearly impossible^64^. Over the past decade, the Warta River has experienced minimal flooding, with low water levels persisting since the last significant floods in 2010 and 2013 (source: Institute of Meteorology and Water Management, Poland). The differentiation found within and among the San, Bug and Narew seems to be mostly due to the IBD, as populations located closer were genetically similar (Sa1 vs. Sa2, Bu1 vs. Bu2, and also Bu3 vs. Na but only to some extent). Relatively free gene flow occurs only along the Vistula, which shares some proportion of its gene pool with the San. This is not surprising as the San River flows into the Vistula in its upper section. The highest genetic variation observed in the middle Vistula is the consequence of its lower degree of transformation, significant gene flow from its tributaries, better landscape connectivity and higher effective population sizes. This is probably the only area where black poplar reproduces generatively, and where seed migration with the river current still plays an important role in the genetic enrichment of downstream populations.

Taking all these into account, at least a few factors have shaped the current genetic structure of black poplar in Poland. Among these, we can name severe genetic bottlenecks in the distant past (which lowered the effective population sizes), restricted gene flow resulting from the transformation of rivers and fragmentation of riparian habitats, as well as virtually no places for generative reproduction. There is also another possible cause of such a situation. Unsuccessful attempts to establish poplar plantations in the 1970s have made black poplar rather unpopular among Polish foresters and breeders. Nevertheless, before the intensive development of the wood industry, black poplar served as a cheap source of wood. This could have led to the elimination of individuals with desirable wood characteristics or those that grew faster or were simply easier to access. Such activities could have additionally increased genetic differentiation.

### Conservation and management of the species genetic resources

Our results indicate the depletion of genetic resources in some populations that necessitates conservation efforts. The effective population sizes were mostly very low. However, based on genetic clustering and AMOVA, we can infer that gene flow occurs both within and between rivers and the level of inbreeding is low. A common rule of “one migrant per generation” is probably still fulfilled. Nevertheless, for some natural populations, this minimum required to maintain connectivity may not be enough^71^. In the present study, populations Od8, Od9 and Wa3 were characterized by lower values of genetic diversity parameters and distinguishable gene pools. This indicates restrictions in gene flow to these populations and warrants conservation actions. The likelihood of extinction due to demographic stochasticity heavily relies on population size. Smaller populations face a higher risk of extinction due to random disturbances like natural disasters^72^. Furthermore, genetic drift depletes allelic diversity more rapidly in smaller populations compared to larger ones^62^. Populations with larger effective sizes have greater chances to maintain a balance between genetic drift and mutations, thus preserving evolutionary potential and adaptability^73^. It has to be noted that almost all populations we analyzed lack opportunities for generative reproduction. Many trees are at a terminal age and their health condition is progressively deteriorating.

The significance of riparian ecosystems makes them one of the priorities for biodiversity conservation strategies, especially considering climate change (see e.g. EU Biodiversity Strategy 2030, EU Floods Directive 2007/60/EC). Unfortunately, the restoration of rivers, including recreation of their natural dynamics, as well as revegetation, is challenging and expensive. There are no provenance trials, nor progeny plantations of black poplar in Poland that could preserve the gene pools of this species and be the source of planting material. Given noticeable genetic structuring, we opt for establishing local clone archives, preferably for all sections of the rivers. If not possible, marginal populations and those with unique gene pools or lower genetic variation should be given priority. In our dataset, these would be Od4, Od8, Od9, Wa3, Vi1, Vi8 and Bu3. Material collection for the establishment of clone archives should be carefully planned. To avoid clones and minimize the relatedness of individuals, the distance between them should be as high as possible, at least 50 m, ideally ~ 100 m. It also has to be taken into account that black poplar is dioecious. Ideally, the sex ratio of clones in the archive should be close to 1:1. We recommend preserving at least 50 distinct genotypes for each section of the rivers analyzed in this study^74^. It is also necessary to identify possible cryptic hybrids using genetic markers.

If local conditions support the long-term survival of young plants, they can be planted using local seed sources as suggested by Wójkiewicz et al.^35^ So far, there exist only six registered seed sources of black poplar, including individuals from population Vi8. As we identified a lot of clones in Vi8, the collection of seeds from this area should be done after the identification of repeated genotypes in the field. It would be reasonable to check the presence of clones, as well as mating patterns, also in the remaining seed sources. Populations characterized by higher genetic variation and effective population size accompanied by relatively low clonality should be registered as new seed sources. As it is hard to fulfill all these criteria, we suggest populations Od5 and Wa2 (after the initial identification of clones), as well as Vi2 and Bu3. The seeds should be collected directly from the trees and preferably from females in good health condition surrounded by the group of male trees to maximize the potential number of pollen donors.

## Conclusions

The results of our study provide essential knowledge about the genetic resources of black poplar in Poland. They also present a compelling argument in the discussion advocating for river renaturation and genetic monitoring, especially concerning keystone species. To date, the genetic studies of black poplar have been limited to Western and Southern Europe and as far east as the Oder valley. The erosion of gene pools observed among populations growing in the regulated parts of the rivers makes it critical to develop a comprehensive conservation strategy for black poplar and to revise the existing seed sources. Ironically, although human activity seems to have been the main driver of the genetic depletion of black poplar, the lack of interest in poplar wood, especially in specimens of questionable quality, enabled the survival of the oldest trees. Nevertheless, without urgent actions, this valuable species will be close to extinction locally, especially in the Warta, Bug and Narew valleys.

## Electronic supplementary material

Below is the link to the electronic supplementary material.

Supplementary Material 1

## Data Availability

Nuclear microsatellite data (PCR products lengths) have been deposited in the Zenodo repository with the accession number 11066213.

## References

[1] Psistaki, K., Tsantopoulos, G. & Paschalidou, A. K. An overview of the role of forests in climate change mitigation. *Sustainability* **16**, 6089 (2024).

[2] Schaberg, P. G., DeHayes, D. H., Hawley, G. J. & Nijensohn, S. E. Anthropogenic alterations of genetic diversity within tree populations: Implications for forest ecosystem resilience. *For.* *Ecol. Manag*. **256**, 855–862 (2008).

[3] Pertoldi, C., Bijlsma, R. & Loeschcke, V. Conservation genetics in a globally changing environment: Present problems, paradoxes and future challenges. *Biodivers. Conserv.* **16**, 4147–4163 (2007).

[4] Pearman, P. B. et al. Monitoring of species’ genetic diversity in Europe varies greatly and overlooks potential climate change impacts. *Nat. Ecol. Evol.* **8**, 267–281 (2024). 38225425 10.1038/s41559-023-02260-0 PMC10857941

[5] Roberts, P., Hamilton, R. & Piperno, D. R. Tropical forests as key sites of the “Anthropocene”: Past and present perspectives. *Proc. Natl. Acad. Sci.* **118**, e2109243118 (2021). 10.1073/pnas.2109243118 PMC8501787 34580229

[6] Pillay, R. et al. Tropical forests are home to over half of the world’s vertebrate species. *Front. Ecol. Environ.* **20**, 10–15 (2022). 35873358 10.1002/fee.2420 PMC9293027

[7] Pielech, R. Plant species richness in riparian forests: Comparison to other forest ecosystems, longitudinal patterns, role of rare species and topographic factors. *For.* *Ecol. Manag*. **496**, 119400 (2021).

[8] Rajpar, M. N., Rajpar, A. H. & Zakaria, M. Riverine forest as a significant habitat to harbor a wide range of bird species. *Braz. J. Biol.* **84**, e256160 (2022). 35137773 10.1590/1519-6984.256160

[9] Nakamura, F. Riparian forests and climate change: Interactive zone of green and blue infrastructure in *Green infrastructure and climate change adaptation: Function, implementation and governance* (ed. Nakamura, F.) 73–91 (Springer Nature, Singapore, 2022).

[10] Šiler, B., Skorić, M. & Mišić, D. General considerations of the European black poplar biology, significance and conservation prospects in *Variability of European black poplar (Populus nigra L.) in the Danube basin* (eds. Tomović, Z. & Vasić, I.) 8–51 (Public Enterprise "Vojvodinašume", 2014).

[11] Lefèvre, F., Légionnet, A., de Vries, S. & Turok, J. Strategies for the conservation of a pioneer tree species, *Populus nigra* L., in Europe. *Genet. Sel. Evol.* **30**, S181–S196 (1998).

[12] Hughes, F. M. R. & Rood, S. B. Allocation of river flows for restoration of floodplain forest ecosystems: A review of approaches and their applicability in Europe. *Environ. Manag.* **32**, 12–33 (2003). 10.1007/s00267-003-2834-8 14703910

[13] Dwire, K. A., Mellmann-Brown, S. & Gurrieri, J. T. Potential effects of climate change on riparian areas, wetlands, and groundwater-dependent ecosystems in the Blue Mountains, Oregon, USA. *Clim. Serv.* **10**, 44–52 (2018).

[14] Pividori, M., Giannetti, F., Barbati, A. & Chirici, G. European forest types: Tree species matrix in *European atlas of forest tree species* (eds. San-Miguel-Ayanz, J., de Rigo, D., Caudullo, G., Houston Durrant, T. & Mauri, A.) e01f162+ (Publications Office of the EU, Luxembourg, 2016).

[15] Vanden Broeck, A. et al. Gene flow between cultivated poplars and native black poplar (*Populus nigra* L.): A case study along the river Meuse on the Dutch-Belgian border. *For.* *Ecol. Manag*. **197**, 307–310 (2004).

[16] Koskela, J., de Vries, S. M. G., Kajba, D. & von Wühlisch, G. *Populus nigra network: Report of the seventh (25-27 October 2001, Osijek, Croatia) and eighth (22-24 May 2003, Treppeln, Germany) meetings* (International Plant Genetic Resources Institute, Rome, 2004).

[17] Benetka, V., Bartáková, I. & Mottl, J. Productivity of *Populus nigra* L. ssp. *nigra* under short-rotation culture in marginal areas. *Biomass Bioenerg*. **23**, 327–336 (2002).

[18] Souch, C. A. & Stephens, W. Growth, productivity and water use in three hybrid poplar clones. *Tree Physiol.* **18**, 829–835 (1998). 12651405 10.1093/treephys/18.12.829

[19] Naiman, R. J., Latterell, J. J., Pettit, N. E. & Olden, J. D. Flow variability and the biophysical vitality of river systems. *Comptes Rendus Geosci.* **340**, 629–643 (2008).

[20] Stettler, R. F., Bradshaw Jr., H. D., Heilman, P. E. & Hinckley, T. M. (eds.) *Biology of Populus and its implications for management and conservation* (NRC Research Press, Ottawa, 1996).

[21] Guilloy-Froget, H., Muller, E., Barsoum, N. & Hughes, F. M. M. Dispersal, germination, and survival of *Populus nigra* L. (Salicaceae) in changing hydrologic conditions. *Wetlands* **22**, 478–488 (2002).

[22] Imbert, E. & Lefèvre, F. Dispersal and gene flow of *Populus nigra* (Salicaceae) along a dynamic river system. *J. Ecol.* **91**, 447–456 (2003).

[23] Pospíšková, M. & Šálková, I. Population structure and parentage analysis of black poplar along the Morava River. *Can. J. For. Res.* **36**, 1067–1076 (2006).

[24] Smulders, M. J. M. et al. Structure of the genetic diversity in black poplar (*Populus nigra* L.) populations across European river systems: Consequences for conservation and restoration. *For.* *Ecol. Manag*. **255**, 1388–1399 (2008).

[25] Jelić, M. et al. Indigenous forests of European black poplar along the Danube River: Genetic structure and reliable detection of introgression. *Tree Genet. Genomes* **11**, 89 (2015).

[26] Čortan, D., Schroeder, H., Šijačić-Nikolić, M., Wehenkel, C. & Fladung, M. Genetic structure of remnant black poplar (*Populus nigra* L.) populations along biggest rivers in Serbia assessed by SSR markers. *Silvae Genet.* **65**, 12–19 (2016).

[27] Çiftçi, A. & Kaya, Z. Genetic diversity and structure of *Populus nigra* populations in two highly fragmented river ecosystems from Turkey. *Tree Genet. Genomes* **15**, 66 (2019).

[28] Çiftçi, A., Yelmen, B., Değirmenci, F. Ö. & Kaya, Z. Impact of biased sex ratio on the genetic diversity, structure, and differentiation of *Populus nigra* (European black poplar). *Botany* **98**, 603–613 (2020).

[29] Barsoum, N., Muller, E. & Skot, L. Variations in levels of clonality among *Populus nigra* L. stands of different ages. *Evol. Ecol.* **18**, 601–624 (2004).

[30] Chenault, N. et al. SSR-based analysis of clonality, spatial genetic structure and introgression from the Lombardy Poplar into a natural population of *Populus nigra* L. along the Loire River. *Tree Genet. Genomes* **7**, 1249–1262 (2011).

[31] Heinze, B. Genetic traces of cultivated hybrid poplars in the offspring of native *Populus nigra* in Austria. *Preslia* **80**, 365–374 (2008).

[32] Smulders, M. J. M. et al. Natural hybridisation between *Populus nigra* L. and *P.* × *canadensis* Moench. Hybrid offspring competes for niches along the Rhine river in the Netherlands. *Tree Genet. Genomes* **4**, 663–675 (2008).

[33] Wójkiewicz, B., Żukowska, W. B., Wachowiak, W. & Lewandowski, A. The genetic assessment of the natural regeneration capacities of black poplar populations in the modern river valley landscapes. *For.* *Ecol. Manag*. **448**, 150–159 (2019).

[34] Lewandowski, A. & Litkowiec, M. Genetic structure of the old black poplar population along the bank of the Vistula River in Poland. *Acta Soc. Bot. Pol.* **86**, 3524 (2017).

[35] Wójkiewicz, B., Lewandowski, A., Żukowska, W. B., Litkowiec, M. & Wachowiak, W. Low effective population size and high spatial genetic structure of black poplar populations from the Oder valley in Poland. *Ann. For. Sci.* **78**, 37 (2021).

[36] Dumolin, S., Demesure, B. & Petit, R. J. Inheritance of chloroplast and mitochondrial genomes in pedunculate oak investigated with an efficient PCR method. *Theor. Appl. Genet.* **91**, 1253–1256 (1995). 24170054 10.1007/BF00220937

[37] Van der Schoot, J., Pospíšková, M., Vosman, B. & Smulders, M. J. M. Development and characterization of microsatellite markers in black poplar (*Populus nigra* L). *Theor. Appl. Genet.* **101**, 317–322 (2000).

[38] Smulders, M. J. M., Van der Schoot, J., Arens, P. & Vosman, B. Trinucleotide repeat microsatellite markers for black poplar (*Populus nigra* L.): Primer note. *Mol. Ecol. Notes*. **1**, 188–190 (2001).

[39] Chapuis, M.-P. et al. Do outbreaks affect genetic population structure? A worldwide survey in *Locusta migratoria*, a pest plagued by microsatellite null alleles. *Mol. Ecol.* **17**, 3640–3653 (2008). 18643881 10.1111/j.1365-294X.2008.03869.x

[40] Matschiner, M. & Salzburger, W. Tandem: Integrating automated allele binning into genetics and genomics workflows. *Bioinformatics* **25**, 1982–1983 (2009). 19420055 10.1093/bioinformatics/btp303

[41] Bailleul, D., Stoeckel, S. & Arnaud-Haond, S. RClone: A package to identify multiLocus clonal lineages and handle clonal data sets in R. *Methods Ecol. Evol.* **7**, 966–970 (2016).

[42] Peakall, R. & Smouse, P. E. GenAlEx 6: Genetic analysis in Excel. Population genetic software for teaching and research. *Mol. Ecol. Notes* **6**, 288–295 (2006). 10.1093/bioinformatics/bts460 PMC3463245 22820204

[43] Peakall, R. & Smouse, P. E. GenAlEx 6.5: Genetic analysis in Excel. Population genetic software for teaching and research—an update. *Bioinformatics* **28**, 2537–2539 (2012). 22820204 10.1093/bioinformatics/bts460 PMC3463245

[44] Goudet, J. Fstat (ver. 2.9.4), a program to estimate and test population genetics parameters. Available from https://www2.unil.ch/popgen/softwares/fstat.htm. Updated from Goudet [1995] (2003).

[45] Chybicki, I. J. & Burczyk, J. Simultaneous estimation of null alleles and inbreeding coefficients. *J. Hered*. **100**, 106–113 (2009). 18936113 10.1093/jhered/esn088

[46] Excoffier, L. & Lischer, H. E. L. Arlequin suite ver 3.5: A new series of programs to perform population genetics analyses under Linux and Windows. *Mol. Ecol. Resour.* **10**, 564–567 (2010). 21565059 10.1111/j.1755-0998.2010.02847.x

[47] Evanno, G., Regnaut, S. & Goudet, J. Detecting the number of clusters of individuals using the software STRUCTURE: A simulation study. *Mol. Ecol.* **14**, 2611–2620 (2005). 15969739 10.1111/j.1365-294X.2005.02553.x

[48] Falush, D., Stephens, M. & Pritchard, J. K. Inference of population structure using multilocus genotype data: Linked loci and correlated allele frequencies. *Genetics* **164**, 1567–1587 (2003). 12930761 10.1093/genetics/164.4.1567 PMC1462648

[49] Puechmaille, S. J. The program STRUCTURE does not reliably recover the correct population structure when sampling is uneven: Subsampling and new estimators alleviate the problem. *Mol. Ecol. Resour.* **16**, 608–627 (2016). 26856252 10.1111/1755-0998.12512

[50] Li, Y.-L., Liu, J.-X. StructureSelector: A web-based software to select and visualize the optimal number of clusters using multiple methods. *Mol. Ecol. Resour.* **18**, 176–177 (2018). 28921901 10.1111/1755-0998.12719

[51] Hardy, O. J., Vekemans, X. SPAGeDi: A versatile computer program to analyse spatial genetic structure at the individual or population levels. *Mol. Ecol. Notes* **2**, 618–620 (2002).

[52] Loiselle, B. A., Sork, V. L., Nason, J. & Graham, C. Spatial genetic structure of a tropical understory shrub, *Psychotria officinalis* (Rubiaceae). *Am. J. Bot.* **82**, 1420–1425 (1995).

[53] Vekemans, X. & Hardy, O. J. New insights from fine-scale spatial genetic structure analyses in plant populations. *Mol. Ecol.* **13**, 921–935 (2004). 15012766 10.1046/j.1365-294x.2004.02076.x

[54] Waples, R. S. & England, P. R. Estimating contemporary effective population size on the basis of linkage disequilibrium in the face of migration. *Genetics* **189**, 633–644 (2011). 21840864 10.1534/genetics.111.132233 PMC3189803

[55] Do, C. et al. NeEstimator v2: Re-implementation of software for the estimation of contemporary effective population size (Ne) from genetic data. *Mol. Ecol. Resour.* **14**, 209–214 (2014). 23992227 10.1111/1755-0998.12157

[56] Cornuet, J. M. & Luikart, G. Description and power analysis of two tests for detecting recent population bottlenecks from allele frequency data. *Genetics* **144**, 2001–2014 (1996). 8978083 10.1093/genetics/144.4.2001 PMC1207747

[57] Peery, M. Z. et al. Reliability of genetic bottleneck tests for detecting recent population declines. *Mol. Ecol.* **21**, 3403–3418 (2012). 22646281 10.1111/j.1365-294X.2012.05635.x

[58] Di Rienzo, A. et al. Mutational processes of simple-sequence repeat loci in human populations. *Proc. Natl. Acad. Sci.* **91**, 3166–3170 (1994). 8159720 10.1073/pnas.91.8.3166 PMC43536

[59] Garza, J. C. & Williamson, E. G. Detection of reduction in population size using data from microsatellite loci. *Mol. Ecol.* **10**, 305–318 (2001). 11298947 10.1046/j.1365-294x.2001.01190.x

[60] Williamson-Natesan, E. G. Comparison of methods for detecting bottlenecks from microsatellite loci. *Conserv. Genet.* **6**, 551–562 (2005).

[61] Hamrick, J. L. & Godt, M. J. W. Effects of life history traits on genetic diversity in plant species. *Philos. Trans. R. Soc. Lond. B Biol. Sci.* **351**, 1291–1298 (1996).

[62] Ellstrand, N. C. & Elam, D. R. Population genetic consequences of small population size: Implications for plant conservation. *Annu. Rev. Ecol. Syst.* **24**, 217–242 (1993).

[63] Arens, P., Coops, H., Jansen, J. & Vosman, B. Molecular genetic analysis of black poplar (*Populus nigra* L.) along Dutch rivers. *Mol. Ecol.* **7**, 11–18 (1998).

[64] Żukowska, W. B., Wójkiewicz, B. & Lewandowski, A. Trunks of multi-stem black poplars may have different genotypes – evidence from the Oder valley in Poland. *Dendrobiology* **86**, 1–7 (2021).

[65] Mazal, L. et al. Fine-scale spatial genetic structure and intra-specific interactions of *Populus nigra* within a natural river corridor along the lower Allier River (France). *Flora* **275**, 151763 (2021).

[66] Macaya-Sanz, D. et al. Causes and consequences of large clonal assemblies in a poplar hybrid zone. *Mol. Ecol.* **25**, 5330–5344 (2016). 27661461 10.1111/mec.13850

[67] Eckert, C. G., Samis, K. E. & Lougheed, S. C. Genetic variation across species’ geographical ranges: The central–marginal hypothesis and beyond. *Mol. Ecol.* **17**, 1170–1188 (2008). 18302683 10.1111/j.1365-294X.2007.03659.x

[68] Meloni, M. et al. Effects of clonality on the genetic variability of rare, insular species: The case of *Ruta microcarpa* from the Canary Islands. *Ecol. Evol.* **3**, 1569–1579 (2013). 23789068 10.1002/ece3.571 PMC3686192

[69] Wei, X. & Jiang, M. Limited genetic impacts of habitat fragmentation in an “old rare” relict tree, *Euptelea pleiospermum* (Eupteleaceae). *Plant. Ecol.* **213**, 909–917 (2012).

[70] Bond, W. J. & Midgley, J. J. Ecology of sprouting in woody plants: The persistence niche. *Trends Ecol. Evol.* **16**, 45–51 (2001). 11146144 10.1016/s0169-5347(00)02033-4

[71] Mills, L. S. & Allendorf, F. W. The one-migrant‐per‐generation rule in conservation and management. *Conserv. Biol.* **10**, 1509–1518 (1996).

[72] Pimm, S. L., Jones, H. L. & Diamond, J. On the risk of extinction. *Am. Nat.* **132**, 757–785 (1988).

[73] Franklin, I. R. Evolutionary changes in small populations in *Conservation biology: An evolutionary-ecological perspective* (eds. Soulé, M. E. & Wilcox, B. M.) 135-149 (Sinauer, Sunderland, 1980).

[74] Bajc, M. et al. (eds.) *Manual for forest genetic monitoring* (Slovenian Forestry Institute, Silva Slovenica Publishing Centre, Ljubljana, 2020).

