# Supplementary material for: Genetic structure and divergence of marginal populations of black poplar (Populus nigra L.) in Poland
Source: Sci Rep. 2025 Jan 23;15:3014. doi: 10.1038/s41598-025-86994-w (PMC11758005; doi:10.1038/s41598-025-86994-w)
Supplement: Supplementary file 1 — Supplementary Material 1 [file 41598_2025_86994_MOESM1_ESM.docx]

**Supplementary Information to “Genetic structure and divergence of marginal populations of black poplar in Poland”**

**Table S1.** Basic genetic variation parameters calculated for each population.

| **River** | **Pop** | **Locality** | **Long** | **Lat** | **N_I_** | **N_L_** | ***R*** | ***A*** | ***A*_R_** | ***A*_E_** | ***A*_P_** | ***H*_O_** | ***H*_E_** |
| --- | --- | --- | --- | --- | --- | --- | --- | --- | --- | --- | --- | --- | --- |
| **Oder** | **Od1** | Kędzierzyn-Koźle | 50.33 | 18.16 | 52 | 46 | 0.88 | 10.89 | 6.49 | 5.50 | 2 | 0.693 | 0.766 |
|  | **Od2** | Brzeg | 51.10 | 17.08 | 53 | 41 | 0.77 | 10.28 | 6.39 | 5.61 | 1 | 0.721 | 0.787 |
|  | **Od3** | Wrocław | 51.10 | 17.08 | 38 | 27 | 0.71 | 9.22 | 6.36 | 5.29 | 2 | 0.688 | 0.776 |
|  | **Od4** | Brzeg Dolny | 51.25 | 16.70 | 89 | 75 | 0.84 | 11.78 | 6.61 | 6.08 | 0 | 0.724 | 0.791 |
|  | **Od5** | Ciechanów | 51.58 | 16.41 | 98 | 51 | 0.52 | 11.72 | 6.70 | 6.07 | 1 | 0.731 | 0.790 |
|  | **Od6** | Bytom Odrzański | 51.74 | 15.82 | 45 | 33 | 0.73 | 9.94 | 6.55 | 5.72 | 1 | 0.729 | 0.794 |
|  | **Od7** | Cigacice | 52.03 | 15.59 | 87 | 68 | 0.78 | 11.33 | 6.53 | 6.10 | 2 | 0.775 | 0.781 |
|  | **Od8** | Kostrzyn nad Odrą | 52.59 | 14.62 | 21 | 17 | 0.81 | 6.33 | 5.06 | 3.93 | 1 | 0.654 | 0.696 |
|  | **Od9** | Gozdowice | 52.76 | 14.32 | 53 | 30 | 0.57 | 6.33 | 4.81 | 3.80 | 3 | 0.668 | 0.708 |
| **Warta** | **Wa1** | Pogorzelica | 52.14 | 17.59 | 66 | 53 | 0.80 | 9.94 | 5.75 | 4.21 | 1 | 0.725 | 0.743 |
|  | **Wa2** | Świątniki | 52.22 | 16.95 | 56 | 37 | 0.66 | 9.56 | 5.96 | 4.60 | 1 | 0.727 | 0.748 |
|  | **Wa3** | Obrzycko | 52.71 | 16.50 | 29 | 27 | 0.93 | 6.89 | 5.23 | 4.22 | 1 | 0.761 | 0.738 |
| **Vistula** | **Vi1** | Tarnobrzeg | 50.55 | 21.64 | 34 | 9 | 0.26 | 6.11 | 5.86 | 4.25 | 1 | 0.753 | 0.757 |
|  | **Vi2** | Puławy | 51.47 | 21.90 | 49 | 44 | 0.90 | 11.94 | 6.96 | 6.37 | 5 | 0.712 | 0.804 |
|  | **Vi3** | Stężyca | 51.58 | 21.78 | 50 | 50 | 1.00 | 12.33 | 7.09 | 6.86 | 4 | 0.749 | 0.812 |
|  | **Vi4** | Kromnów | 52.38 | 20.35 | 32 | 32 | 1.00 | 9.94 | 6.42 | 5.50 | 3 | 0.683 | 0.790 |
|  | **Vi5** | Kamion | 52.37 | 20.20 | 55 | 45 | 0.82 | 11.72 | 6.98 | 6.73 | 2 | 0.753 | 0.808 |
|  | **Vi6** | Toruń | 53.01 | 18.62 | 37 | 33 | 0.89 | 10.50 | 6.57 | 5.89 | 2 | 0.764 | 0.796 |
|  | **Vi7** | Solec Kujawski | 53.09 | 18.22 | 33 | 21 | 0.64 | 8.50 | 6.14 | 5.20 | 1 | 0.730 | 0.765 |
|  | **Vi8** | Mątowy Wielkie | 54.03 | 18.89 | 31 | 13 | 0.42 | 7.72 | 6.41 | 5.04 | 3 | 0.664 | 0.765 |
| **San** | **Sa1** | Ulanów | 50.50 | 22.26 | 48 | 46 | 0.96 | 10.67 | 6.30 | 5.62 | 1 | 0.763 | 0.776 |
|  | **Sa2** | Stalowa Wola | 50.58 | 22.08 | 47 | 47 | 1.00 | 11.33 | 6.61 | 5.98 | 3 | 0.751 | 0.795 |
| **Bug** | **Bu1** | Terespol | 52.11 | 23.62 | 35 | 34 | 0.97 | 8.50 | 5.61 | 4.43 | 0 | 0.693 | 0.750 |
|  | **Bu2** | Mielnik | 52.32 | 23.05 | 19 | 18 | 0.95 | 7.33 | 5.74 | 4.66 | 0 | 0.683 | 0.768 |
|  | **Bu3** | Kamieńczyk | 52.61 | 21.53 | 73 | 73 | 1.00 | 9.78 | 5.53 | 4.34 | 1 | 0.712 | 0.732 |
| **Narew** | **Na** | Bindużka | 52.79 | 21.34 | 31 | 30 | 0.97 | 8.89 | 6.13 | 5.29 | 1 | 0.703 | 0.767 |

Pop – population acronym; Long – longitude; Lat – latitude; N_I_ – number of individuals; N_L_ – number of clonal lineages; *R* – genotypic richness (N_L_/N_I_); *A* – mean number of alleles; *A*_R_ – mean rarefied allelic richness; *A*_E_ – mean effective number of alleles; *A*_P_ – number of private alleles; *H*_O_ – mean observed heterozygosity; *H*_E_ – mean expected heterozygosity.

**Table S2.** Pairwise *F*_ST_ (below diagonal) and *R*_ST_ (above diagonal) values among the studied populations. Values that are not significant (p > 0.05) are written in italics.

|  | **Od1** | **Od2** | **Od3** | **Od4** | **Od5** | **Od6** | **Od7** | **Od8** | **Od9** | **Wa1** | **Wa2** | **Wa3** | **Vi1** | **Vi2** | **Vi3** | **Vi4** | **Vi5** | **Vi6** | **Vi7** | **Vi8** | **Sa1** | **Sa2** | **Bu1** | **Bu2** | **Bu3** | **Na** |
| --- | --- | --- | --- | --- | --- | --- | --- | --- | --- | --- | --- | --- | --- | --- | --- | --- | --- | --- | --- | --- | --- | --- | --- | --- | --- | --- |
| **Od1** |  | .025 | .022 | .077 | .057 | .055 | .052 | .050 | .081 | .060 | .068 | .048 | .061 | .036 | .039 | .109 | .050 | .043 | .057 | .048 | .042 | .067 | .076 | .104 | .071 | .036 |
| **Od2** | .036 |  | .032 | .109 | .090 | .103 | .097 | .111 | .115 | .087 | .065 | .117 | .072 | .078 | .069 | .152 | .082 | .050 | .101 | .054 | .124 | .153 | .144 | .154 | .118 | .044 |
| **Od3** | .040 | .024 |  | .038 | .037 | .030 | .047 | .025 | .044 | .041 | .043 | .078 | .121 | .033 | .044 | .106 | .058 | .026 | .038 | *.028* | .088 | .088 | .071 | .106 | .138 | *.009* |
| **Od4** | .045 | .042 | .039 |  | .022 | .017 | .024 | .040 | .057 | .044 | .053 | .072 | .205 | .034 | .031 | .094 | .070 | .071 | .049 | .068 | .079 | .094 | .051 | .123 | .149 | .032 |
| **Od5** | .034 | .031 | .025 | .034 |  | *.004* | .014 | .039 | .053 | .045 | .047 | .058 | .158 | .028 | .019 | .073 | .042 | .055 | .039 | .048 | .075 | .085 | .048 | .097 | .114 | .021 |
| **Od6** | .043 | .041 | .035 | .027 | .026 |  | *.003* | .020 | .022 | .068 | .058 | .046 | .225 | .031 | .034 | .112 | .073 | .079 | .065 | .069 | .062 | .109 | .070. | .131 | .146 | .033 |
| **Od7** | .046 | .054 | .045 | .040 | .028 | .031 |  | .038 | .041 | .051 | .044 | .037 | .164 | .031 | .019 | .074 | .045 | .056 | .036 | .048 | .051 | .085 | .060 | .112 | .110 | .021 |
| **Od8** | .078 | .070 | .078 | .070 | .060 | .064 | .066 |  | .056 | .059 | .074 | .049 | .185 | .036 | .058 | .101 | .082 | .070 | .054 | .071 | .066 | .078 | .058 | .110 | .153 | .035 |
| **Od9** | .071 | .077 | .077 | .061 | .056 | .062 | .056 | .080 |  | .116 | .115 | .084 | .252 | .073 | .081 | .177 | .121 | .106 | .115 | .110 | .085 | .143 | .111 | .171 | .203 | .064 |
| **Wa1** | .080 | .073 | .058 | .070 | .064 | .078 | .061 | .114 | .109 |  | .045 | .090 | .105 | .047 | .032 | .050 | .035 | .037 | .032 | .059 | .107 | .064 | .065 | .120 | .132 | .022 |
| **Wa2** | .067 | .064 | .072 | .067 | .067 | .067 | .070 | .089 | .106 | .058 |  | .118 | .147 | .053 | .045 | .055 | .047 | .034 | .045 | *.018* | .132 | .145 | .115 | .106 | .129 | *.006* |
| **Wa3** | .089 | .106 | .107 | .104 | .095 | .092 | .085 | .142 | .139 | .086 | .097 |  | .181 | .067 | .055 | .146 | .093 | .124 | .116 | .112 | .059 | .118 | .110 | .195 | .135 | .074 |
| **Vi1** | .063 | .059 | .062 | .064 | .061 | .057 | .061 | .090 | .107 | .085 | .083 | .117 |  | .132 | .109 | .124 | .075 | .070 | .131 | .088 | .177 | .124 | .202 | .192 | .125 | .091 |
| **Vi2** | .031 | .037 | .037 | .035 | .027 | .032 | .038 | .061 | .067 | .067 | .067 | .083 | .034 |  | *.005* | .050 | .019 | .022 | .018 | *.019* | .027 | .054 | .018 | .042 | .045 | *.010* |
| **Vi3** | .030 | .035 | .039 | .034 | .027 | .030 | .031 | .063 | .069 | .058 | .060 | .067 | .031 | .006 |  | .045 | .011 | .027 | .027 | .027 | .041 | .062 | .039 | .085 | .049 | *.011* |
| **Vi4** | .043 | .049 | .055 | .057 | .044 | .046 | .050 | .078 | .091 | .062 | .062 | .082 | .051 | .029 | .019 |  | .015 | .042 | .040 | *.029* | .124 | .088 | .093 | .076 | .097 | .035 |
| **Vi5** | .036 | .034 | .042 | .042 | .027 | .031 | .041 | .064 | .071 | .061 | .061 | .076 | .038 | .014 | .013 | .022 |  | .013 | .022 | *.003* | .078 | .060 | .060 | .074 | .052 | *.013* |
| **Vi6** | .041 | .041 | .044 | .044 | .040 | .040 | .051 | .082 | .083 | .060 | .070 | .085 | .051 | .021 | .022 | .028 | .019 |  | .007 | *.002* | .088 | .065 | .059 | .047 | .086 | *.000* |
| **Vi7** | .057 | .061 | .060 | .065 | .051 | .063 | .059 | .104 | .102 | .069 | .079 | .097 | .060 | .034 | .039 | .045 | .036 | .034 |  | *.018* | .079 | .041 | .026 | .051 | .115 | *.000* |
| **Vi8** | .061 | .051 | .059 | .064 | .057 | .062 | .070 | .086 | .098 | .081 | .091 | .094 | .059 | .018 | .030 | .041 | .021 | .023 | .031 |  | .100 | .105 | .090 | .064 | .071 | *.000* |
| **Sa1** | .039 | .058 | .060 | .048 | .043 | .044 | .045 | .082 | .083 | .072 | .077 | .086 | .043 | .016 | .018 | .036 | .026 | .033 | .047 | .045 |  | .062 | .048 | .105 | .070 | .063 |
| **Sa2** | .049 | .060 | .061 | .057 | .050 | .045 | .052 | .089 | .090 | .087 | .095 | .089 | .043 | .021 | .021 | .037 | .030 | .034 | .041 | .037 | .026 |  | .031 | .102 | .127 | .068 |
| **Bu1** | .075 | .070 | .077 | .066 | .064 | .068 | .066 | .099 | .100 | .087 | .083 | .112 | .078 | .048 | .046 | .062 | .044 | .066 | .065 | .074 | .059 | .066 |  | .061 | .129 | .042 |
| **Bu2** | .050 | .071 | .076 | .069 | .062 | .053 | .072 | .093 | .104 | .105 | .089 | .116 | .071 | .038 | .046 | .053 | .044 | .044 | .069 | .068 | .048 | .059 | .054 |  | .118 | .047 |
| **Bu3** | .073 | .088 | .096 | .084 | .078 | .082 | .086 | .105 | .113 | .106 | .116 | .120 | .087 | .048 | .052 | .066 | .040 | .048 | .074 | .055 | .060 | .060 | .087 | .076 |  | .085 |
| **Na** | .075 | .070 | .075 | .075 | .065 | .072 | .071 | .098 | .110 | .081 | .095 | .099 | .075 | .038 | .044 | .049 | .032 | .031 | .046 | .030 | .052 | .047 | .072 | .074 | .049 |  |

**Figure S1.** Mantel test results.


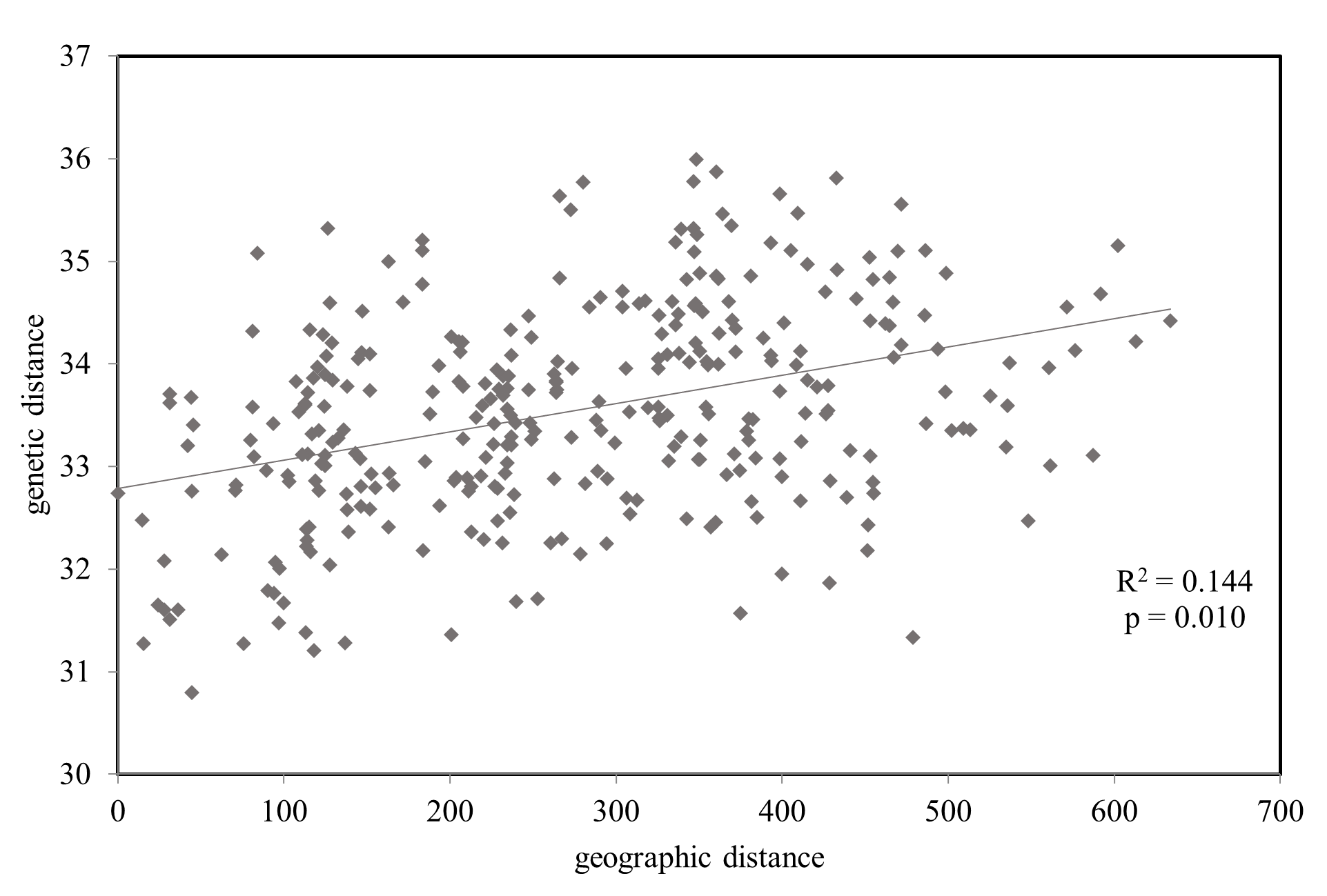


**Figure S2.** Correlograms of the spatial genetic structure (SGS) of each population. Loiselle’s kinship coefficient is plotted for ten discrete distance classes with respective 95% confidence intervals (dotted lines). All Sp values are significant at p < 0.05.

**
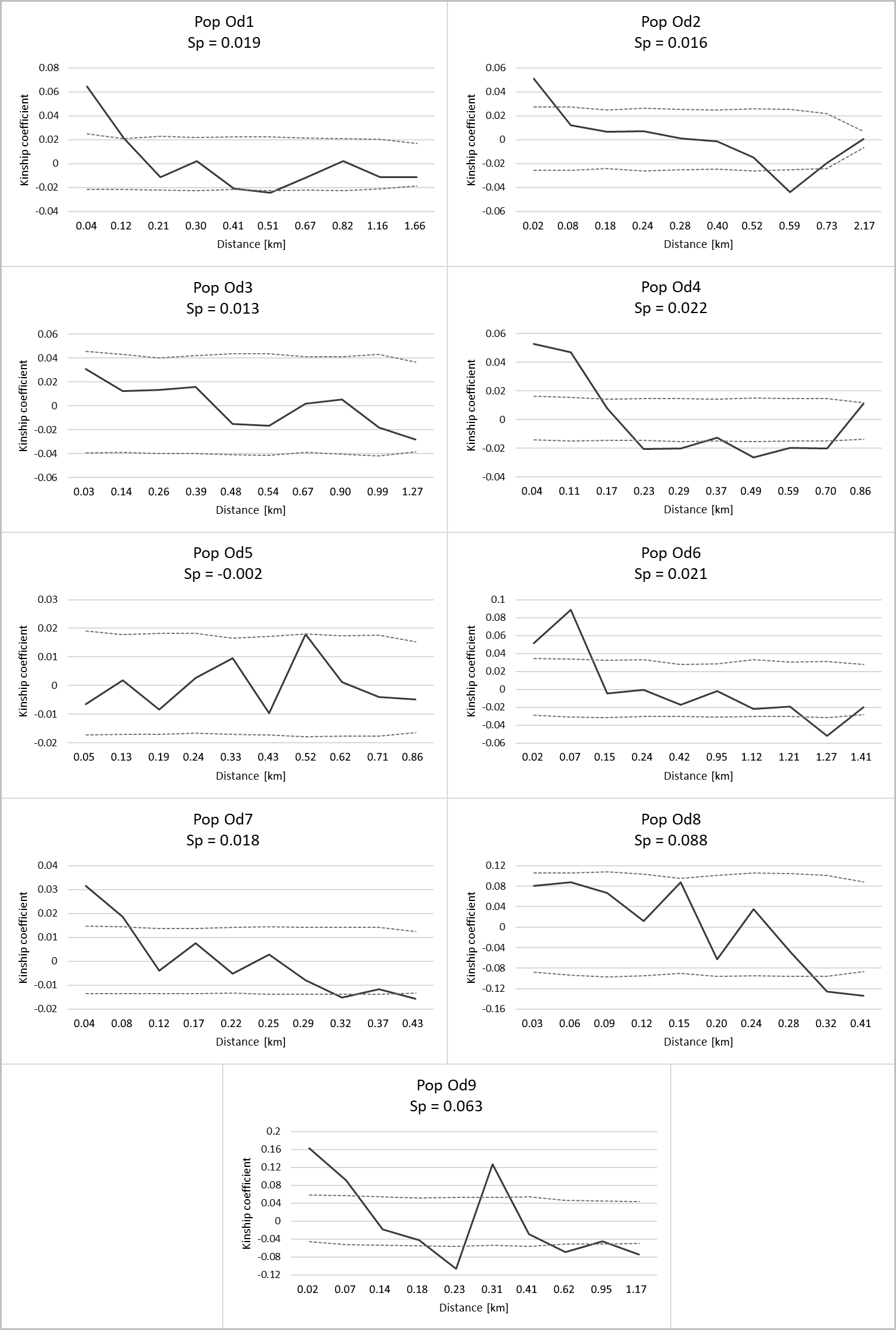
**

**
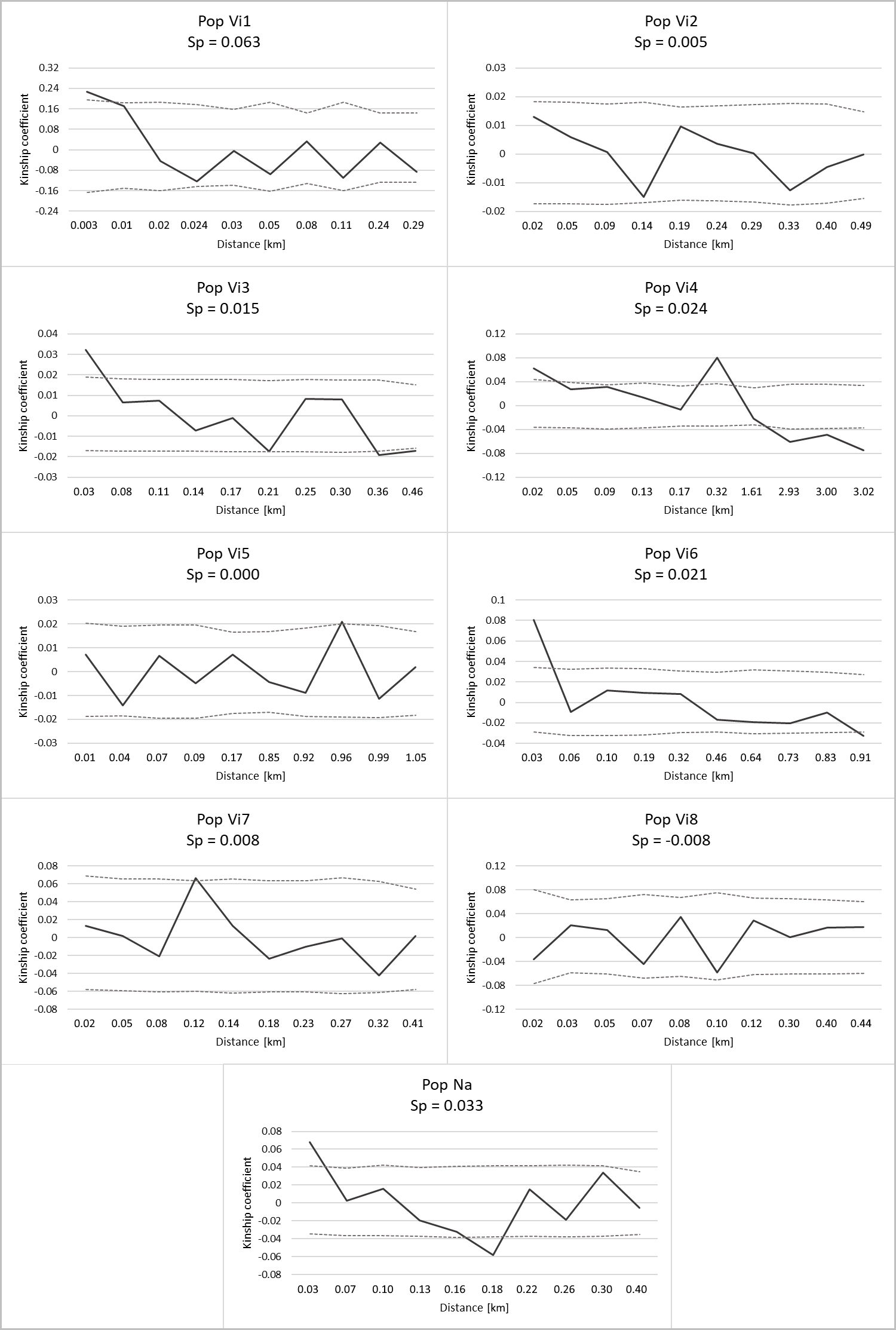
**

**
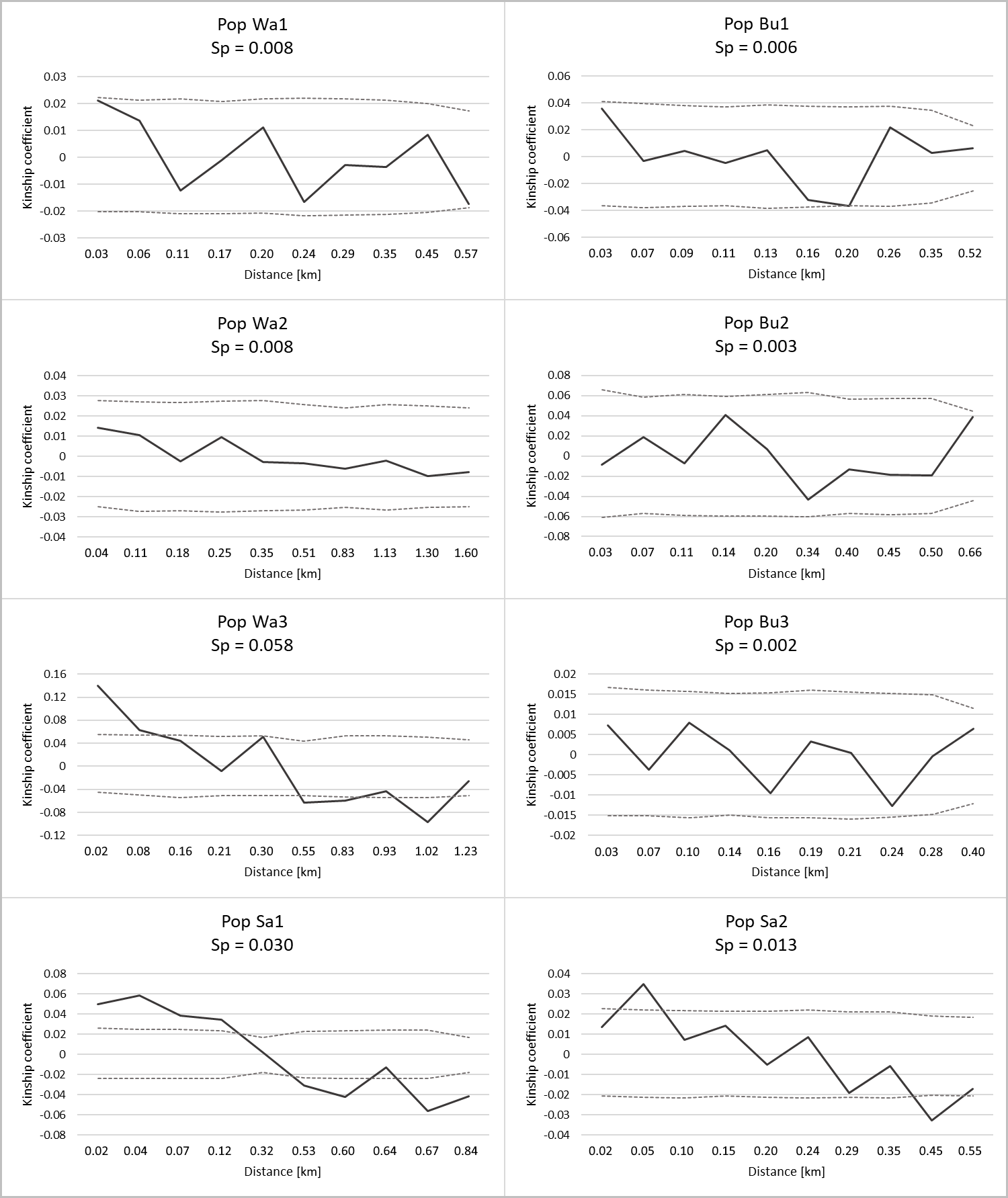
**
